# Hybrid between *Danio rerio* female and *Danio nigrofasciatus* male produces aneuploid sperm with limited fertilization capacity

**DOI:** 10.1371/journal.pone.0233885

**Published:** 2020-05-29

**Authors:** Mitsuru Endoh, Fumika Shima, Miloš Havelka, Rei Asanuma, Etsuro Yamaha, Takafumi Fujimoto, Katsutoshi Arai

**Affiliations:** 1 Laboratory of Aquaculture Genetics and Genomics, Division of Marine Life Science, Faculty and Graduate School of Fisheries Sciences, Hokkaido University, Hakodate, Hokkaido, Japan; 2 Research Fellow of Japan Society for the Promotion of Science, Tokyo, Japan; 3 Nanae Fresh-Water Station, Field Science Center for Northern Biosphere, Hokkaido University, Nanae, Hokkaido, Japan; University Hospital of Münster, GERMANY

## Abstract

In the *Danio* species, interspecific hybridization has been conducted in several combinations. Among them, only the hybrid between a zebrafish (*D*. *rerio*) female and a spotted danio (*D*. *nigrofasciatus*) male was reported to be fertile. However, beyond these investigations, by means of reproductive biology, gametes of the hybrid have also not been investigated genetically. For this study, we induced a hybrid of the *D*. *rerio* female and *D*. *nigrofasciatus* male in order to study its developmental capacity, reproductive performance and gametic characteristics. Its hybrid nature was genetically verified by polymerase chain reaction-restriction fragment length polymorphism (PCR-RFLP) analysis of the *rhodopsin* gene. Almost all the hybrids (36/37) were males, and only one was female. Developing oocytes were observed in the hybrid female, but ovulated eggs have not been obtained thus far. Microscopic observation revealed various head sizes of sperm in the hybrid males. Flow cytometry showed that the hybrid males generated aneuploid sperm with various ploidy levels up to diploidy. In backcrosses between *D*. *rerio* females and hybrid males, fertilization rates were significantly lower than the control *D*. *rerio*, and most resultant progeny with abnormal appearance exhibited various kinds of aneuploidies ranging from haploidy to triploidy, but only one viable progeny, which survived more than four months, was triploid. This suggested the contribution of fertile diploid sperm of the hybrid male to successful fertilization and development.

## Introduction

Much research has been conducted into disclosing the biological characteristics of fish hybrids [1–3]. Generally, fish hybrids between closely related species can survive until adulthood, and some are fertile, generating functional gametes and producing a viable second generation. However, those between distantly related species, belonging to a different genus or family, find it difficult to survive; and even if they do, most resultant hybrids lack reproductive capacity.

Examples in salmonids include the interspecific hybrids *Salvelinus namaycush* (female) x *S*. *fontinalis* (male) [4], *S*. *fontinalis* x *S*. *alpinus* [5], *S*. *fontinalis* x *S*. *pluviu*s [6], and *Oncorhynchus masou* x *O*. *rhodurus* [6] that were reported to produce viable progenies after back-crossings to parental species and/or inter-crossings between F_1_ hybrids. By contrast, inter-generic hybrids died before the beginning of feeding and free swimming (swim-up) in many combinations of parental species, and most survivors exhibited undeveloped gonads [7]. Although the inter-generic hybrids *Salmo trutta* x *Salvelinus fontinalis*, *S*. *fontinalis* x *O*. *masou* and *S*. *fontinalis* x *O*. *nerka* generated functional gametes, they could not produce viable backcross progeny due to abnormal embryonic development [6]. Similar situations have been found in fish hybrids belonging to other families [1].

Hybridization sometimes gives rise to atypical reproduction modes. Unreduced egg formation is found in interspecific hybrids between *Salmo salar* and *S*. *trutta* [8], as well as in hybrids between *Oryzias latipes* and *O*. *curvinotus* [9], in which triploid progenies were produced by back-crossing between unreduced eggs and sperm of a parental species [9]. Similar occurrences of unreduced gametes are also reported in cyprinid hybrids between crucian carp (*Carassius auratus*) and common carp (*Cyprinus carpio*) [10]. Natural clonal individuals of dojo loach (*Misgurnus anguilllicaudatus*) produce isogenic diploid eggs, which develop by gynogenesis [11] and such atypical reproduction is caused by a past hybridization event between two genetically diverse groups [12, 13]. In marine *Hexagrammos* species, hemi-clonal reproduction by hybridogenesis has been recently discovered, and this phenomenon is also caused by a past hybridization event [14–16].

Although there is a large number of studies on hybridization in fish, the cellular and molecular mechanisms underlying developmental and reproductive success or failure of hybrids are not yet well understood. Hybrid inviability could be explained by aneuploidies due to chromosome eliminations in the course of embryonic development in salmonid hybrids [17, 18], but the molecular mechanisms of the expression of embryonic abnormality have not been disclosed yet. There are relatively few studies on the mechanisms of hybrid sterility in fish [19, 20] or in other vertebrates [21–24]. According to these studies, irregular meiotic configurations, including univalent and multivalent chromosomes, are due to the failure of synapses and are considered to be the primary cause of gonadal or gametic sterility. Recently, mitotic arrest of primordial germ cells (PGCs), followed by a disappearance of germ cells, has been reported as another cause of sterility in inter-generic marine fish hybrids between *Nibea* and *Pennahia* [25]. A similar phenomenon has been reported as a lack of germ cells [26] or the presence of “neuter” hybrid individuals without gonads [7]. Thus far, our knowledge about hybrid sterility is limited, and its mechanism is still unclear.

To deepen our understanding of the developmental and reproductive characteristics of hybrids, more intensive analysis using model fish is necessary. The zebrafish is a useful experimental animal because of its small body size, ease of rearing and breeding, and short life span. In the genus *Danio*, containing zebrafish (*Danio rerio*, or *D*. *rerio*) and its related species, hybridization has not been well studied compared with the high number of studies using zebrafish in other scientific fields [27]. Interspecific hybridization among *Danio* specie has been performed in several combinations. Among them, hybrids between *D*. *rerio* female and other *Danio* males (*D*. *albolineatus*, *D*. *kerri* and *D*. *dangila*) were reported to be sterile [28]. A hybrid between *D*. *rerio* and *D*. *albolineatus* was used for the sterile host in surrogate production because sterile hybrids do not generate endogenous gametes, and thus only transplanted exogenous germ cells are able to differentiate into functional gametes in the gonads of germ-line chimera [29].

Among the *Danio* hybrids examined thus far, the hybrid between the *D*. *rerio* female and a *D*. *nigrofasciatus* male was the only fertile one [28]. Successful reproduction of this hybrid might be explained by the close phylogenetic relationship, inferred from the sequences of *12S* and *16S rDNA* [30] and *rhodopsin* [31]. Although this hybrid could produce gametes, it could not produce viable progeny by back-crossings to parental species [28]. In the aforementioned report, the reproductive characteristics were not described in detail, so the reason for the failure in producing a second generation was inconclusive. For our own research, we produced a *D*. *rerio* female x *D*. *nigrofasciatus* male hybrid in order to re-examine its developmental and reproductive characteristics in as much detail as possible. We also studied the gonadal and gametic characteristics of the hybrid individuals, and then attempted to produce a second generation by back-crossing sperm from hybrid males to the eggs of *D*. *rerio* females.

## Materials and methods

### Ethics

This study was carried out in accordance with the Guide for the Care and Use of Laboratory Animals in Hokkaido University. The experimental procedures were approved by the Institutional Animal Care and Use Committee of National University Corporation Hokkaido University (approval number 29–3).

### Interspecific hybridization and backcross

Wild-type strain *D*. *rerio* and *D*. *nigrofasciatus* were bought by aquarium shop and kept in the laboratory, maintained under a 14-h light and 10-h dark photoperiod at 28.5°C. Ovulated eggs from *D*. *rerio* and sperm from *D*. *rerio* and *D*. *nigrofasciatus* were collected as previously described [32]. Three *D*. *rerio* females and three *D*. *nigrofasciatus* males were used for hybridization. Interspecific hybrid was induced by artificial fertilization of *D*. *rerio* eggs with *D*. *nigrofasciatus* sperm. We also induced a *D*. *rerio* control group by artificial fertilization of *D*. *rerio* eggs with *D*. *rerio* sperm. A hybrid between *D*. *nigrofasciatus* females and *D*. *rerio* males could not be induced because ovulated eggs from *D*. *nigrofasciatus* were not obtained.

After one-year post fertilization, we tried to collect sperm and eggs from the hybrid between the *D*. *rerio* female and *D*. *nigrofasciatus* male. To collect the sperm, the abdomen of the hybrid specimen was squeezed under anesthesia with tricaine methanesulfonate (MS222) (Sigma-Aldrich, St. Louis, USA). Sperm collected from the hybrid males was diluted with artificial seminal plasma (Kurokura solution) [33], and used for backcross fertilization with *D*. *rerio* eggs. The hybrids that did not produce sperm were paired with *D*. *rerio* males to induce ovulation because these hybrids were presumed to be female.

### Developmental capacity

To investigate the developmental capacity of the hybrid between the *D*. *rerio* female and *D*. *nigrofasciatus* male, and the backcross progeny from the hybrid sperm fertilized with *D*. *rerio* eggs, we measured the fertilization rate, survival rate and rate of individuals with normal external appearance (normal rate). The fertilization rate was calculated as the number of cleaved eggs relative to the total number of eggs used at the 8–128 cell stage. The survival rate was calculated by the number of surviving embryos relative to the number of cleaved eggs at the hatching stage, i.e., 48–72 hours post fertilization. The normal rate was calculated by the number of externally normal larvae relative to the number of survival larvae at hatching stage.

### Genetic analysis

Hybrid nature was genetically verified by polymerase chain reaction-restriction fragment length polymorphism (PCR-RFLP) analysis of the *rhodopsin* gene. To perform hybrid identification, we have developed an assay for the species identification of *D*. *rerio* and *D*. *nigrofasciatus*.

Primers were designed as (RhodopsinF, 5’-GCGGCCTACATGTTCTTCCTCATC-3’), and (RhodopsinR, 5’-TGTAGATGCAGGGGTTGTAGACGG-3’) by Primer 3, implemented in Geneious 11.0. The sequences of *rhodopsin* gene were referred from the NCBI database of the *D*. *rerio* (AF105152, AF109368, JQ614147 and NM131084) and *D*. *nigrofasciatus* (JQ614145, JQ614146 and HM223909). A 799 bp of the *rhodopsin* gene region was amplified using polymerase chain reaction (PCR). PCR was performed with a 10 μL mixture containing 1.0 μL of the DNA sample, 1.2 μL of double-distilled water (DDW), 5.0 μL of 2x Buffer for KOD FX Neo (TOYOBO, Osaka, Japan), 2.0 μL of 2 mM deoxynucleotide triphosphate (dNTP) (TOYOBO), 0.2 μL of KOD FX Neo (TOYOBO), and 0.3 μL of each 10 μM primer. The PCR conditions were as follows: initial denaturation 2 m at 94°C, 30 cycles of 10 s at 98°C, 30 s at 64°C, 30 s at 68°C. PCR amplicon was digested with the DNA restriction enzyme *Bmg*T120 I (TaKaRa, Shiga, Japan) at 37°C for 16 h. The PCR-RFLP fragments were electrophoresed and stained with ethidium bromide.

### Investigation of gonadal sex

The gonadal sex of the hybrid was investigated by observation of the gonad appearance using a stereo microscope (MZ16F-RCFL, LEICA, Wetzlar, Germany) and histological analysis. At approximately two years post fertilization, 37 hybrid individuals were sacrificed under anesthesia with overdose of MS222 and dissected. The gonads of the hybrids were observed and collected under a stereoscopic microscope, and then fixed overnight with Bouin’s fixative. The fixed gonads were embedded in resin or paraffin, and histologically sectioned at 2- or 6-μm thickness, respectively. The sections were stained with hematoxylin and eosin.

### Observation of spermatozoa

Spermatozoa from ten hybrid males and three *D*. *rerio* males were observed by phase contrast microscopy (DM5500B, LEICA). The diameter of the spermatozoa head was measured using Image J. Before back-crossings, sperm activity was confirmed under microscope in a bright field. Sperm activation was induced by mixing with tap water.

### Ploidy analysis

The relative DNA content of fin-clips and sperm of *D*. *rerio* and the hybrid, as well as the surviving larvae from all the backcross groups, were measured by flow cytometry (CyFlow; Partec GmbH, Münster, Germany), according to a previously described procedure [32]. In a ploidy analysis of the sperm, Kurokura solution [33] was removed by centrifugation. The collected sperm was incubated in 100 μL of solution A (CyStain DNA 2step) (Partec GmbH) that extracted nuclei for 20 m. Then, 20 μL of the solution was mixed with 800 μL of solution B (CyStain DNA 2step) (Partec GmbH) that contained 4’, 6-diamidino-2-phenylindole (DAPI), and stained extracted nuclei, and analyzed by flow cytometry. The ploidy and aneuploidy were estimated based on relative DNA content, when the 2C DNA content of fin-clips obtained from diploid *D*. *rerio* was set as 2n diploid standard.

### Statistical analysis

The data for developmental ability in the induction of the hybrid and backcross fertilization were shown as mean ± standard deviation, and were analyzed by Kruskal-Wallis test, followed by a Scheffe’s test (*p* < 0.05). A part of the data for developmental ability in backcross fertilization of Exps. 4 and 5 were analyzed by Welch's t test (*p* < 0.05).

## Results

### Developmental capacity

There was no significant difference between the hybrid and the control in fertilization and survival rates (Table 1). The normal rate of the hybrid derived from the sperm of *D*. *nigrofasciatus* male No. 2 was significantly lower than other hybrid groups and the control group (Table 1).

**Table 1:** Developmental capacity of the hybrid between *Danio rerio* female and *D*. *nigrofasciatus* male.

Crosses		No. of eggs used	Fertilization (%)	Survival at hatching stage (%)	Normal larvae (%)
Female x Male					
*D*. *rerio*	*D*. *rerio*	114 ± 32	48.1 ± 33.8 ^a^	83.9 ± 11.9 ^a^	72.0 ± 25.5 ^a^
	*D*. *nigrofasciatus* No. 1	141 ± 20	47.1 ± 39.1 ^a^	90.7 ± 0.6 ^a^	69.1 ± 5.1 ^ab^
	*D*. *nigrofasciatus* No. 2	130 ± 27	11.4 ± 4.5 ^a^	87.5 ± 6.5 ^a^	28.6 ± 9.7 ^b^
	*D*. *nigrofasciatus* No. 3	155 ± 81	41.3 ± 25.1 ^a^	88.6 ± 4.3 ^a^	48.1 ± 12.0 ^ab^

Each value is the mean ± SD of the triplicate.

Different superscript letters in each column indicate significant differences as determined by Kruskal–Wallis test and Scheffe’s test (*P* < 0.05).

### Genetic analysis

**Fig 1:**
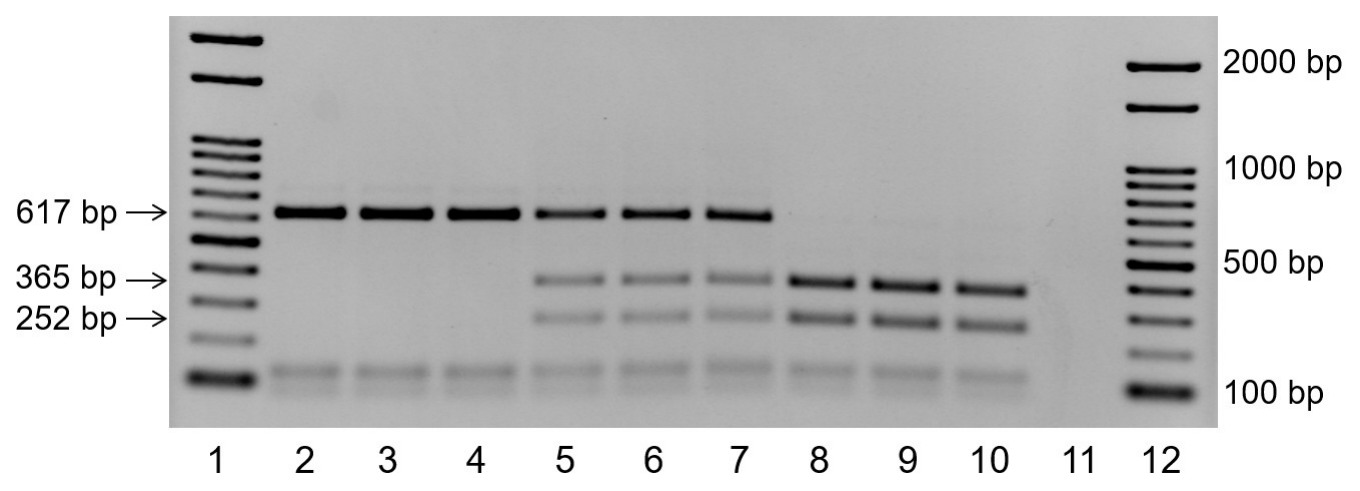
PCR-RFLP analysis of *rhodopsin* gene. (Lanes 1 and 12) Molecular size marker. (Lanes 2–4) *D*. *rerio* gave a single fragment of 617 bp. (Lanes 5–7) The hybrids between *D*. *rerio* female and *D*. *nigrofasciatus* male possessed three fragments of 617, 365 and 252 bp. (Lanes 8–10) *D*. *nigrofasciatus* gave two fragments of 365 and 252 bp. (Lane 11) Negative control.

To confirm whether hybrid samples inherited the genome set from both *D*. *rerio* and *D*. *nigrofasciatus*, a PCR-RFLP analysis of the *rhodopsin* gene was performed (Fig 1). The female *D*. *rerio* possessed 617 bp species-specific fragments. In contrast, the male *D*. *nigrofasciatus* had species-specific 252- and 365-bp fragments. All hybrid samples possessed paternally derived 252, 365, and maternally derived 617 bp PCR-RFLP fragments. Therefore, all the hybrid samples used for our experiments where confirmed to possess genome sets from *D*. *rerio* and *D*. *nigrofasciatus*.

### Gonadal sex of the hybrid

**Fig 2:**
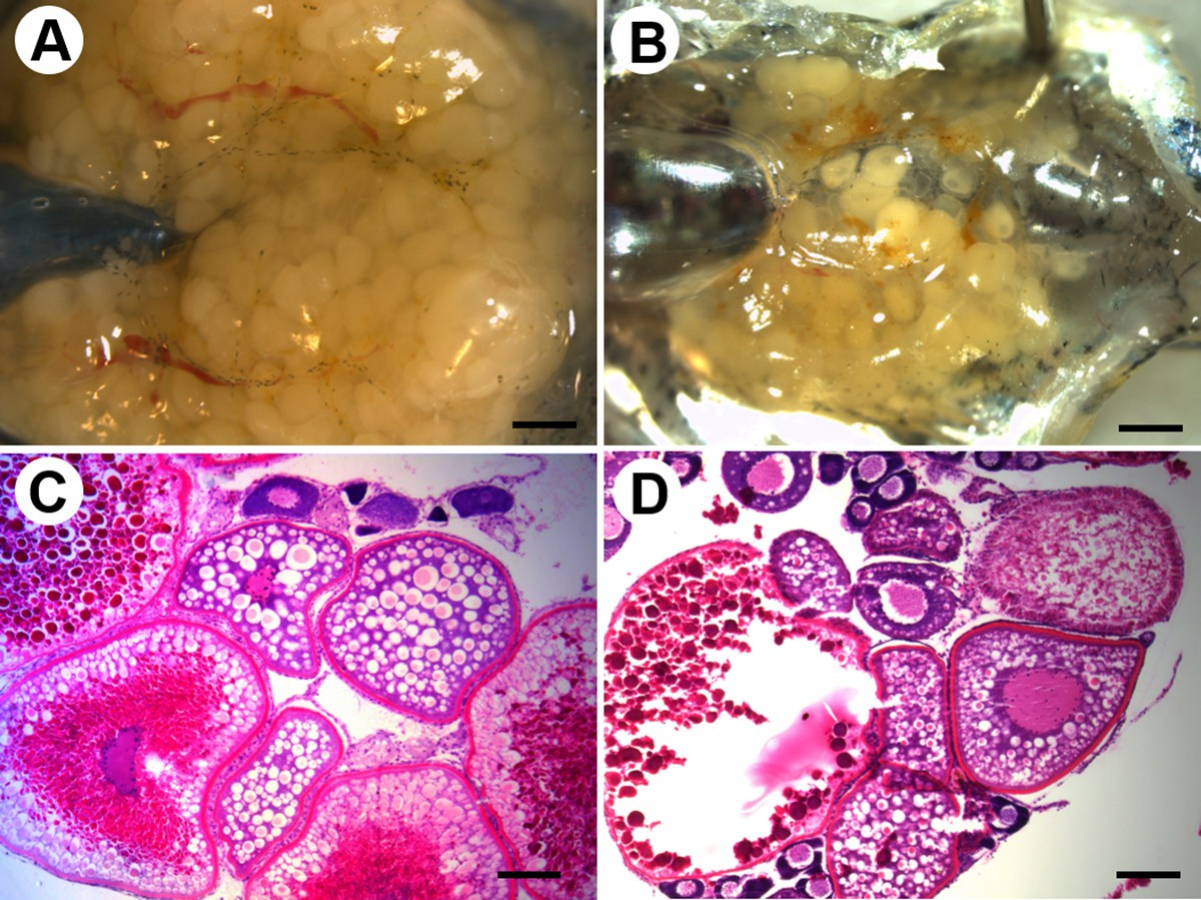
External appearance and histological images of ovaries. (A, C) *D*. *rerio*, (B, D) the hybrid between a *D*. *rerio* female and a *D*. *nigrofasciatus* male. Scale bars indicate 1 mm (A and B) and 100 μm (C and D).

To investigate the gonadal development of the hybrid samples, the *D*. *rerio* and the hybrids were dissected at two years post fertilization. The gonadal sex and fertility of a total of 37 hybrid individuals are shown in Table 2. The ovary of the *D*. *rerio* was filled with oocytes (Fig 2A), but the number of visible oocytes in the hybrid ovary was fewer and the ovary itself was smaller than the *D*. *rerio* (Fig 2B). Developing oocytes in the cortical alveolus (yolk vesicle) stage and vitellogenesis (yolk globule) stage were observed in the hybrid ovary as well as the *D*. *rerio* (Fig 2C and 2D). However, ovulated eggs could not be obtained from the hybrid female.

**Table 2:** Sex ratio and reproductive characteristics of the hybrid between *D*. *rerio* female and *D*. *nigrofasciatus* male at two years post fertilization.

Female	Males	
	Aneuploid sperm	No sperm
1	10	26

**Fig 3:**
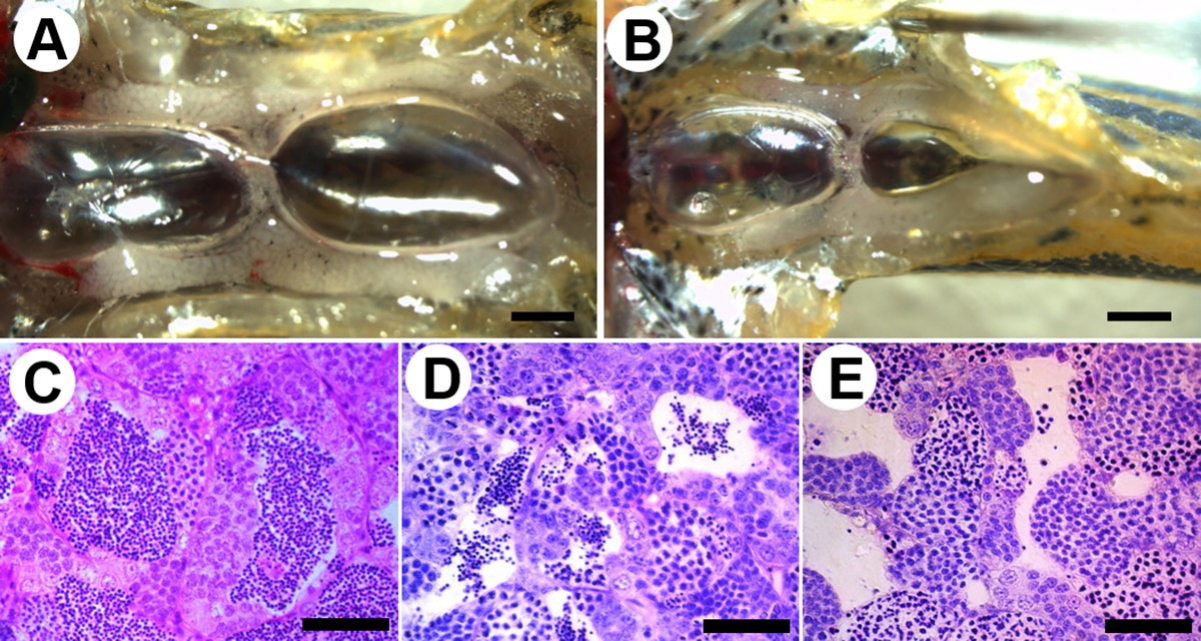
External appearance and histological images of testis. (A, C) *D*. *rerio*, (B, D and E) the hybrid between *D*. *rerio* female and *D*. *nigrofasciatus* male. (C) Lumen in the control *D*. *rerio* testis was fully filled with mature spermatozoa. (D) A small number of mature spermatozoa was observed in the lumen of the hybrid male that generated aneuploid sperm. (E) There were no mature spermatozoa in the lumen of hybrid males that did not produce sperm. Scale bars indicate 1 mm (A and B) and 50 μm (C–E).

The external appearance of the hybrid testis was morphologically identical to the *D*. *rerio* male (Fig 3A and 3B), and there was no difference in external appearance between the hybrid male that produced sperm and the hybrid male that did not. In the *D*. *rerio* testis, each lumen was filled with mature spermatozoa (Fig 3C). In contrast, there were fewer mature spermatozoa in the lumen of the hybrid males than the *D*. *rerio* (Fig 3D). In addition, some hybrid males that did not produce sperm had no observable spermiation or mature spermatozoa (Fig 3E). Ten hybrid males produced sperm, while the remaining 26 did not (Table 2).

### Sperm morphology and ploidy level

The concentration of hybrid sperm was lower than the *D*. *rerio* sperm. However, almost all hybrid spermatozoa had motility immediately after activation by tap water (concentration and velocity were not measured).

**Fig 4:**
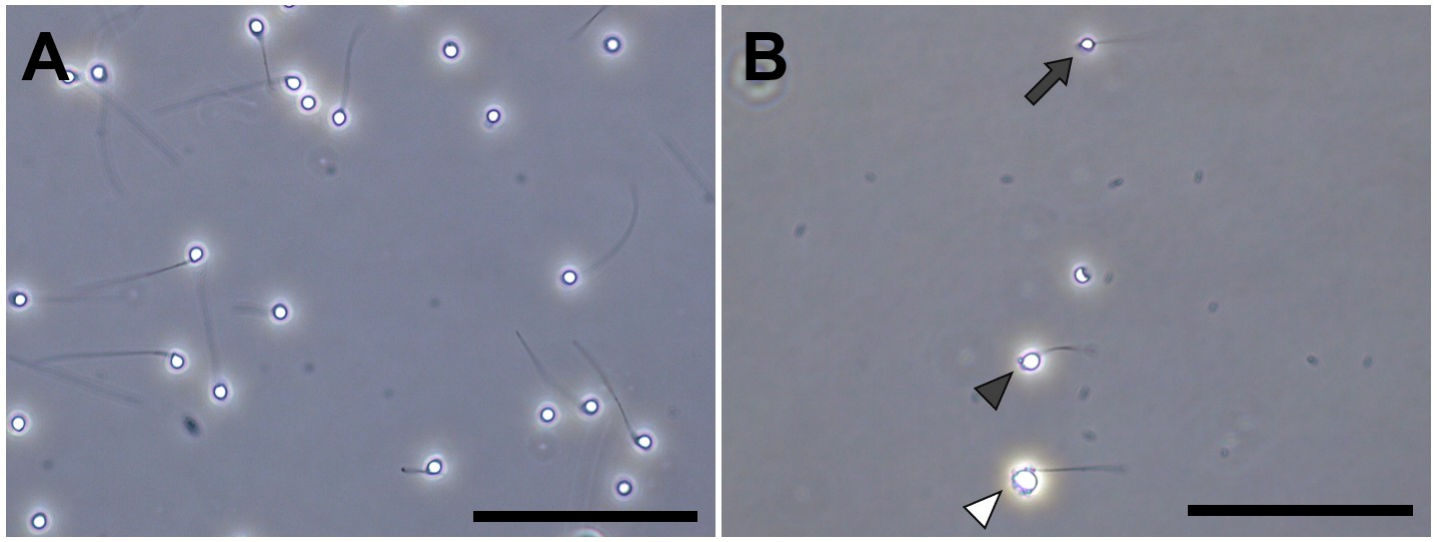
Morphology of spermatozoa. (A) *D*. *rerio*, (B) the hybrid between *D*. *rerio* female and *D*. *nigrofasciatus* male. Spermatozoa morphology of *D*. *rerio* and the hybrid between *D*. *rerio* female and *D*. *nigrofasciatus* male. Head diameter of hybrid spermatozoa were 2.12 μm (solid arrow), 3.23 μm (solid arrowhead) and 4.41 μm (open arrowhead). Scale bars indicate 50 μm.

**Fig 5:**
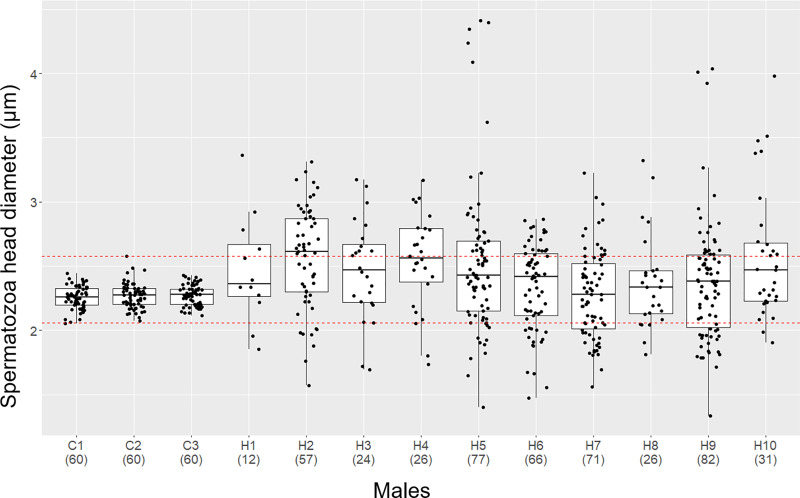
Box plot showing spermatozoa head diameter (μm). (C1–C3) *D*. *rerio* males, (H1–H10) hybrids between *D*. *rerio* female and *D*. *nigrofasciatus* male. Red dashed lines delimit the smallest and the largest spermatozoa head diameter in *D*. *rerio* males. Each dot represents one measured spermatozoon. Number of analyzed spermatozoa in brackets.

The spermatozoa of the *D*. *rerio* had a generally consistent head size (Fig 4A)–the average spermatozoa head diameter of the *D*. *rerio* males was 2.26 ± 0.09–2.27 ± 0.10 μm. In contrast, the hybrid sperm exhibited various head sizes (Fig 4B). The average head diameter of the hybrid spermatozoa (2.28 ± 0.34–2.57 ± 0.40 μm) was almost the same, or slightly larger than the *D*. *rerio* (Fig 5). Among the 472 hybrid spermatozoa measured, 88 were smaller than the smallest one in *D*. *rerio* (2.05 μm) while 158 were larger than the largest one in *D*. *rerio* (2.57 μm). The smallest head diameter in hybrids was 1.34 μm, and the largest was 4.41 μm. Detail distribution of spermatozoa head diameter within the sperm population of each individual can be seen in Fig 5.

**Fig 6:**
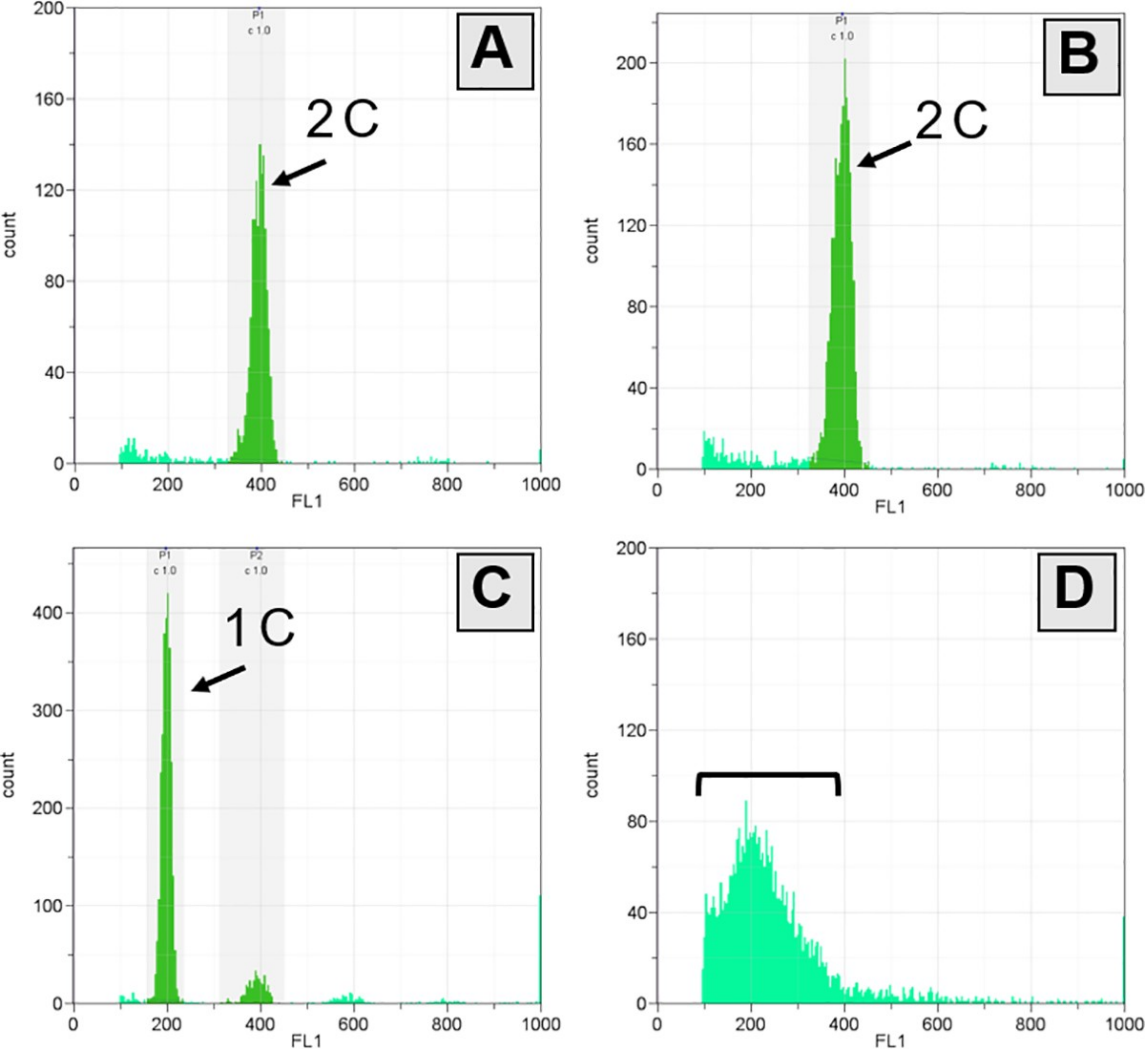
Relative DNA contents measured by flow cytometry of fin-clips (A, B) and sperm (C, D). (A, C) *D*. *rerio* diploid control, (B, D) the hybrid between *D*. *rerio* female and *D*. *nigrofasciatus* male. Square bracket indicates range of aneuploid spermatozoa up to about diploidy.

Fin-clips from all the hybrid samples showed the same diploid (2C) DNA content (Fig 6B) as the diploid *D*. *rerio* had as standard (Fig 6A). The relative DNA content of *D*. *rerio* sperm was half that of the diploid control (Fig 6C) implying that only haploid spermatozoa were produced. Despite the somatic cells of the hybrid being the same diploid as *D*. *rerio*, flow cytometry showed that the spermatozoa of the hybrid males were aneuploidies, with various ploidy levels up to diploidy (Fig 6D). The histogram of the relative DNA contents showed normal distribution with haploidy as the mode.

### Backcross progeny

In back-crossings, fertilization rates were always significantly lower than the *D*. *rerio* controls (Table 3). The hybrid male No. 2 had a higher fertilization ability than the other hybrid males (Nos. 1, 3 and 4). In back-crossings with sperm from hybrid males No. 7 and No. 9, the survival rate at hatching stage was significantly lower than the control. However, there was no significant difference in survival rates between the control and the other back-crossings (sperm from hybrid males Nos. 1–6, 8 and 10). Normal rates of all back-crossings were significantly lower than those of the control. Three larvae with normal external appearance arose from back-crossings of males Nos. 2, 5 and 10.

**Table 3:** Developmental capacity of backcross progeny between *D*. *rerio* female and the hybrid males.

Experiment no.	Crosses		No. of eggs used	Fertilization (%)	Survival at hatching stage (%)	Normal larvae (%)
	Female x Male					
Exp. 1	*D*. *rerio*	*D*. *rerio*	88 ± 28	63.0 ± 7.9 ^a^	98.2 ± 1.6 ^a^	93.9 ± 0.8 ^a^
		Hybrid No. 1	106 ± 30	5.4 ± 2.1 ^c^	72.2 ± 25.5 ^a^	0.0 ± 0.0 ^b^
		Hybrid No. 2	134 ± 67	24.1 ± 3.3 ^b^	69.6 ± 8.7 ^a^	1.9 ± 3.2 ^b^
		Hybrid No. 3	90 ± 40	3.7 ± 0.2 ^c^	86.7 ± 23.1 ^a^	0.0 ± 0.0 ^b^
		Hybrid No. 4	109 ± 68	8.9 ± 2.8 ^c^	59.7 ± 8.7 ^a^	0.0 ± 0.0 ^b^
Exp. 2	*D*. *rerio*	*D*. *rerio*	57 ± 25	71.6 ± 16.0 ^a^	79.2 ± 24.3 ^a^	62.7 ± 30.8 ^a^
		Hybrid No. 5	114 ± 17	18.4 ± 2.4 ^b^	60.3 ± 14.2 ^a^	2.3 ± 4.5 ^b^
		Hybrid No. 6	116 ± 10	2.3 ± 1.6 ^b^	52.5 ± 41.1 ^a^	0.0 ± 0.0 ^b^
Exp. 3	*D*. *rerio*	*D*. *rerio*	119 ± 33	73.5 ± 12.6 ^a^	90.4 ± 3.6 ^a^	91.7 ± 4.5 ^a^
		Hybrid No. 7	171 ± 42	2.0 ± 1.1 ^b^	40.0 ± 27.1 ^b^	0.0 ± 0.0 ^b^
		Hybrid No. 8	162 ± 51	4.3 ± 1.7 ^b^	75.1 ± 20.4 ^ab^	0.0 ± 0.0 ^b^
Exp. 4	*D*. *rerio*	*D*. *rerio*	87 ± 24	60.4 ± 15.5 ^a^	96.0 ± 5.3 ^a^	84.1 ± 8.9
		Hybrid No. 9	130 ± 56	3.6 ± 1.4 ^b^	28.1 ± 19.2 ^b^	0.0 ± 0.0
Exp. 5	*D*. *rerio*	*D*. *rerio*	69 ± 15	75.7 ± 17.2 ^a^	93.6 ± 6.6 ^a^	93.7 ± 1.7 ^a^
		Hybrid No.10	155 ± 37	2.7 ± 2.4 ^b^	45.2 ± 43.1 ^a^	5.6 ± 9.6 ^b^

Each value is the mean ± SD of the triplicates (in Exps. 1, 4 and 5) or four replicates (in Exps. 2 and 3).

Different superscript letters in each column of Exps. 1, 2 and 3 indicates significant differences as determined by Kruskal–Wallis test and Scheffe’s test (*P* < 0.05).

Different superscript letters in each column of Exps. 4 and 5 indicates significant differences as determined by Welch’s t test (*P* < 0.05).

**Fig 7:**
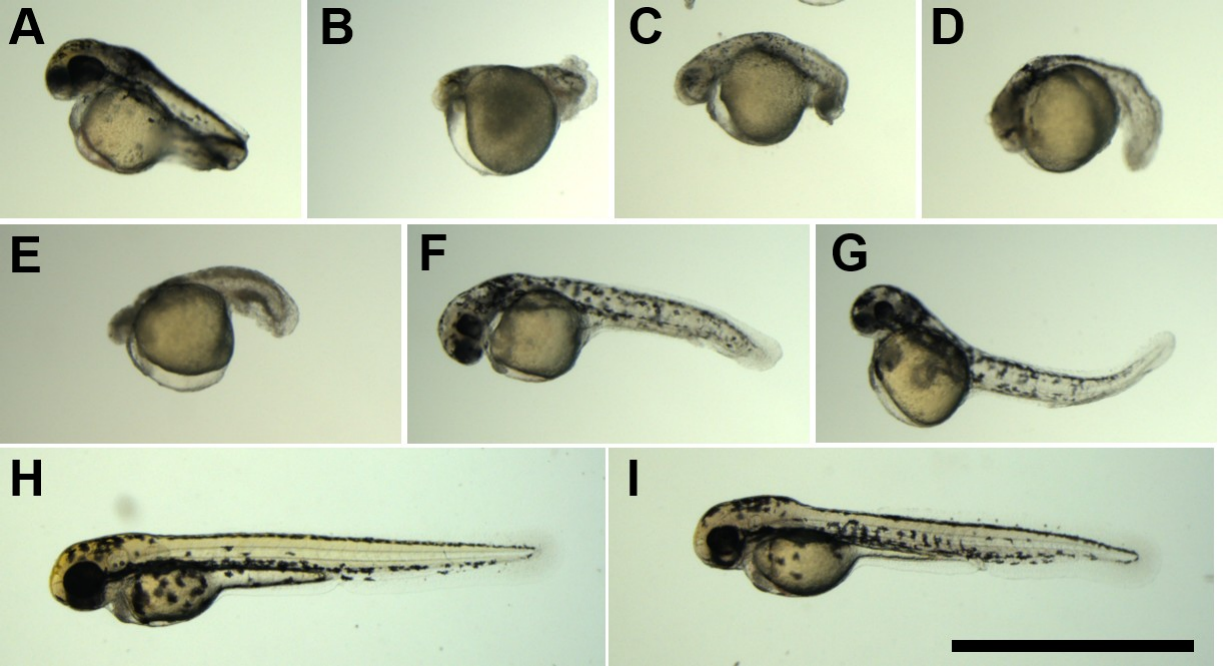
External appearance of backcross progeny with various ploidy or aneuploidy levels. (A) 1n larva, (B) 1.3n larva, (C) 1.5n larva, (D) 1.8n larva, (E) 2n larva, (F) 2.3n larva, (G) 2.5n larva, (H) 3n larva without blood circulation, and (I) 2.5n larva with normal appearance. All larvae were progeny from fertilization between *D*. *rerio* female and the interspecific hybrid male at 48 hours post fertilization. Scale bar indicates 1 mm.

**Fig 8:**
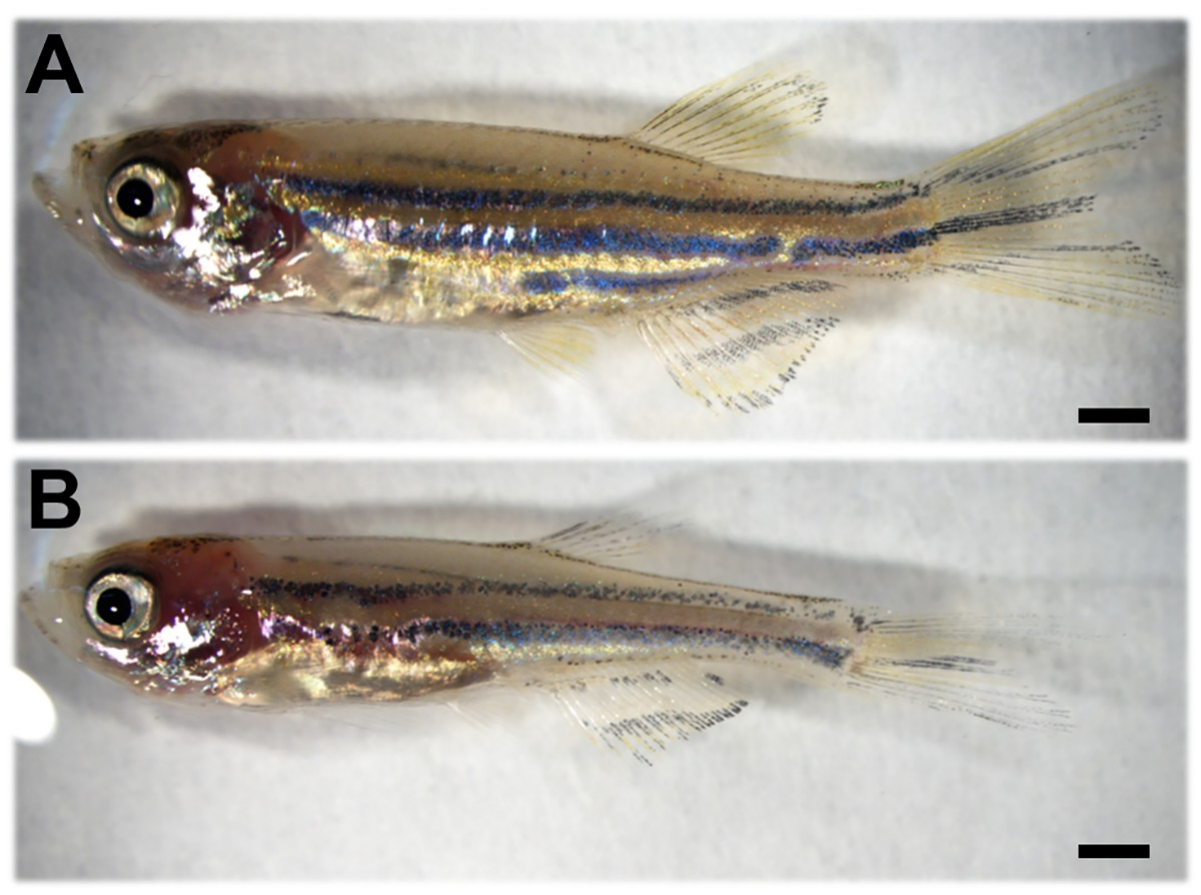
Two-month-old backcross progeny from fertilization between *D*. *rerio* female and the hybrid male. (A) *D*. *rerio* diploid control, (B) surviving triploid backcross progeny with normal appearance. Scale bars indicate 1 mm.

Progeny obtained from the backcrosses showed various aneuploidies, approximately ranging from haploidy to triploidy (Table 4). Haploid (1n) larvae exhibited an abnormality known as haploid syndrome (Fig 7A). Hypodiploid (1.3n, 1.5n and 1.8n) larvae exhibited severe abnormalities with deficient formation of eyes and body axis (Fig 7B, 7C and 7D). All diploid (2n) larvae that originated from back-crossings also exhibited abnormal appearance, despite the euploid (Fig 7E). In the hyperdiploid 2.3n (Fig 7F) and2.5n (Fig 7G), the external abnormalities were less severe than in the hypodiploid larvae. Externally normal larvae without blood circulation were also classified as abnormal (Fig 7H). As the ploidy level increased, the external appearance tended to be closer to normal. Backcross progeny with normal appearance from the hybrid males No. 2, 5 and 10, were 2.5n (Fig 7I), 2.9n and 3n, respectively. Only the triploid backcross progeny survived more than four months post fertilization (Fig 8B), the same as in the control diploid progeny (Fig 8A) from normal fertilization between *D*. *rerio* female and male.

**Table 4:** Ploidy level based on relative DNA contents of progeny obtained from backcross between *D*. *rerio* female and hybrid males.

Crosses		External appearance	No. of larvae for FCM	Ploidy status estimated by relative DNA content (C)					
Female x Male				1n	1.1–1.9n	2n	2.1–2.9n	3n	Mosaic
*D*. *rerio*	*D*. *rerio*	Normal	68	0	0	68	0	0	0
		Abnormal	30	7	1	20	1	1	0
*D*. *rerio*	Hybrid 1	Abnormal	48	0	10	11	24	2	1^a^
*D*. *rerio*	Hybrid 2	Normal	1	0	0	0	1	0	0
		Abnormal	49	2	15	4	27	0	0
*D*. *rerio*	Hybrid 3	Abnormal	8	0	4	1	3	0	0
*D*. *rerio*	Hybrid 4	Abnormal	17	0	6	2	9	0	0
*D*. *rerio*	Hybrid 5	Normal	1	0	0	0	1	0	0
		Abnormal	63	4	17	5	36	1	0
*D*. *rerio*	Hybrid 6	Abnormal	13	0	6	0	7	0	0
*D*. *rerio*	Hybrid 7	Abnormal	11	0	7	0	4	0	0
*D*. *rerio*	Hybrid 8	Abnormal	24	3	6	2	13	0	0
*D*. *rerio*	Hybrid 9	Abnormal	3	0	0	1	2	0	0
*D*. *rerio*	Hybrid 10	Normal	1	0	0	0	0	1	0
		Abnormal	7	0	2	2	3	0	0

Each value is the total of triplicates or four replicates.

^a^ 3.0n-4.0n mosaic

## Discussion

### Gonadal sex of the hybrids

In this study, the gonadal sex of the hybrids was almost all male. Among the bitterlings (*Rhodeus* sp.), a species of cyprinid with female heterogametic genetic sex determination (female ZW, male ZZ), physiological (gonadal) sex is also biased to males in artificial hybrids [34]. Although the genetic sex of the hybrid should be female (ZW), many individuals had sex-reversed into physiological males, presumably due to unpredicted interactions between the Z and W chromosomes from different species [34]. An excessive number of males also appeared among triploid flatfish hybrids between plaice and flounder [35]. A similar male-biased sex ratio was also observed in induced triploid zebrafish (*D*. *rerio*) [36]. In *D*. *rerio*, it has been found that testis formation is promoted by a depletion of PGCs, together with the accompanying changes in gene expression [37]. Mitotic arrest of PGCs was recently detected in the genital ridges of sciaenid hybrids between *Nibea mitsukurii* and *Pennahia argentata*, and this phenomenon was highlighted as a major cause of hybrid sterility [25], although a similar disappearance of germ cells has been inconclusively described as “neuter” in some salmonid and cyprinid hybrids [7, 26, 38]. In our study, the gonadal sex of the hybrids between *D*. *rerio* females and *D*. *nigrofasciatus* males is presumably biased to male by depleting, or not increasing, the PGC count, due to a certain amount of compatibility between *D*. *rerio* and *D*. *nigrofasciatus* genomes in undifferentiated gonads.

### Aneuploid sperm

Some of the hybrid males did not produce sperm, while others produced aneuploid spermatozoa with various ploidy, ranging up to diploidy with an approximate mode of haploidy. This result was supported by the size variation of the spermatozoa head diameter. There was no difference in the average diameter of spermatozoa between the hybrid and *D*. *rerio* males. However, spermatozoa with less than 2 μm or greater than 3 μm were observed only in hybrids. It was considered that the spermatozoa head sizes changed depending on the difference in aneuploidy level. Similarly, spermatozoa head size and size of erythrocytes nuclear area were found to be positively correlated with ploidy and chromosome number in polyploid sturgeons [39–41]

Aneuploid spermatozoa have been observed in triploid males of dojo loach [42, 43], grass carp [44], rainbow trout [45] and a hybrid between rainbow trout and brook trout [46]. Triploid grass carp, rainbow trout and salmonid hybrids produced an average ploidy of 1.5n spermatozoa. However, induced triploid dojo loach produced an average of 1.3n spermatozoa which is lower than the theoretical ploidy of 1.5n. Natural triploid loach *M*. *anguillicaudatus*, from the wild population in China, produced aneuploid sperm ranging from 1.2n to 2.2n [43]. It has been suggested that aneuploid sperm is formed by equal segregation of bivalents and random segregation of univalents in triploid germ cells [42, 47]. The formation of irregular configurations, including univalents and multivalents, was detected both in female and male triploid dojo loach [48, 49]. In interspecific hybrid fishes, cytogenetic studies on meiotic configurations are scarce. However, in an induced inter-group hybrid between group A and group B, which are almost equal to the interspecific genetic distance in dojo loach, it was recently reported that both bivalents and univalent were formed in spermatocytes [20]. Such coexistence of bivalents and univalents presumably causes the formation of aneuploid spermatozoa, owing to a random segregation of univalents in the interspecific hybrids. The results suggest that a failure of synapsis should occur in hybrids. Thus, in hybrids between *D*. *rerio* and *D*. *nigrofasciatus*, univalent chromosomes may be randomly separated to generate aneuploid spermatozoa. Another possibility is the completely random segregation of all chromosomes to form various aneuploidies up to diploidy. In this case, the chromosomes cannot find any homologous counterpart to pair with for the formation of bivalents, and all such univalents may segregate at random to form various kinds of aneuploidies, up to diploidy, with a theoretical mode on the haploidy. To conclude on the cytological mechanisms for the production of aneuploid gametes in backcross progeny, further precise observation of meiotic configurations is necessary in future studies.

### Backcross progeny

Among the 257 backcross individuals analyzed for ploidy level, only three of the progeny had a morphologically normal appearance. Only one backcross individual survived till four-month. Most backcross progeny exhibited various kinds of abnormalities, possibly due to aneuploidies, and they did not survive beyond the beginning of feeding and swim-up stage. However, it is known that aneuploid individuals are not always inviable. The 3.5n progeny between a rainbow trout (*Oncorhynchus mykiss*) female and a triploid hybrid *O*. *mykiss* x *S*. *fontinalis* male survived until eight months post fertilization [46]. Viable aneuploid (hypertriploid) progeny were also detected in dojo loach, when diploid eggs from a tetraploid loach were fertilized with the 1.3n aneuploid sperm of induced triploid males [42]. Viable hyperdiploid dojo loach were also reported when regular haploid eggs were fertilized with aneuploid sperm of induced triploid [50]. These results suggest that individual fish comprising at least a diploid genome (two sets of chromosomes) and extra chromosomes, i.e., hyperdiploid and hypotriploid, could survive.

Thus, it is suggested that the viability of aneuploidies may be closely related to the genomic constitution of aneuploid sperm. In the case of aneuploid sperm by triploid male, viable aneuploid progeny could be obtained by aneuploid sperm comprising at least haploid genome (one set of chromosomes) and extra chromosomes fertilized with a haploid egg. However, for this study, it was not necessary to include a set of normal genomes in the aneuploid sperm of the hybrid between *D*. *rerio* and *D*. *nigrofasciatus* when considering the results of the ploidy analysis of sperm and backcross progeny, because the hybrids were diploid (not triploid). Despite the euploidy, all the diploid backcross progeny from the haploid eggs of the *D*. *rerio* female fertilized with the haploid sperm of the hybrid males exhibited an abnormal appearance. In addition, the 1.3n, 1.5n and 1.8n hypodiploid embryos that were likely fertilized with 0.3n, 0.5n and 0.8n sperm derived from hybrid were detected. The level of abnormality in these hypodiploid progeny was more severe than that of the haploid progeny, suggesting harmful effects by paternal extra chromosomes on embryonic development. It was not possible to determine whether haploid progeny was due to spontaneous gynogenesis or fertilization with nullisomic (0n) sperm from the hybrids. In hyperdiploid larvae such as 2.3n and 2.5n, and triploid larvae, the extent of abnormalities was much less, and the external appearance was near normal or normal. As the number of chromosomes contained in the hybrid sperm nucleus increased, the number of sperm with the chromosomes necessary for normal embryonic development increased. However, at the same time, the number of chromosomes that caused abnormalities also increased in the hybrid sperm. A four-month-old survivor of backcross progeny was triploid that was presumably produced by diploid sperm derived from the hybrid fertilized with a *D*. *rerio* haploid egg. It is still unclear whether these diploid sperm were formed by random chromosome segregation or by other mechanisms, such as premeiotic duplication.

### Possibility of hybridization in nature and hybrid speciation

In nature, interspecific hybridization occurs frequently in plants, but might be seen also between closely related animal species [51]. From an evolutionary biological perspective, hybridization is one of important process of speciation and contributes to the expansion of biodiversity. In fact, hybrid speciation has been reported not only in plants [52], but also in amphibia [53] and in fish species [54]. On the other hand, hybridization causes admixture of non-native genes to gene pools of native species that may result in the dilution and/or an irreparable loss of adaptability. This is especially relevant to endangered species and it may significantly disrupt conservation efforts. The negative effect of interspecific hybridization may be intensified even further by backcross of fertile hybrids to negative species resulting in “genetic cleaning” of hybrid genotypes, and thus loss of the evidence for ancestral hybridization [55]. Hybridization between *D*. *rerio* female and *D*. *nigrofasciatus* male can occur easily even in mixed breeding aquarium [56]. We showed that their hybrids are fully viable and they are not completely sterile, as they produce sperm with fertilization ability. At present, natural habitats of these two species are separated by the Arakan Mountains [57] preventing hybridization in nature. Once human disturbance or loss of physical barrier occurs, introgression might occur due to natural hybridization events between these two species. Highly reduced reproductive success of hybrid due to the low progeny survival is likely.

## Conclusion

The zebrafish (*D*. *rerio*) x spotted danio (*D*. *nigrofasciatus*) hybrid males produced aneuploid sperm ranged up to diploidy, with fertilization ability. However, most backcross progeny by the hybrid sperm fertilized with *D*. *rerio* eggs could not survive, due to abnormalities caused by aneuploidy. The possibility that some backcross progeny from hybrid sperm may reach adulthood could not be fully ruled out.

## Supporting information

S1 Raw images Original image for gel of PCR-RFLP (Fig 1). (Lanes 1 and 12) Molecular size marker. (Lanes 2–4) *D*. *rerio* gave a single fragment of 617 bp. (Lanes 5–7) The hybrids between *D*. *rerio* female and *D*. *nigrofasciatus* male possessed three fragments of 617, 365 and 252 bp. (Lanes 8–10) *D*. *nigrofasciatus* gave two fragments of 365 and 252 bp. (Lane 11) Negative control.
(PDF)

S1 File ARRIVE guidelines checklist. (PDF)
