# Supplementary figures and images for: Hybrid between Danio rerio female and Danio nigrofasciatus male produces aneuploid sperm with limited fertilization capacity
Source: PLoS One. 2020 May 29;15(5):e0233885. doi: 10.1371/journal.pone.0233885 (PMC7259755; doi:10.1371/journal.pone.0233885)

617 bp →  
365 bp →  
252 bp →

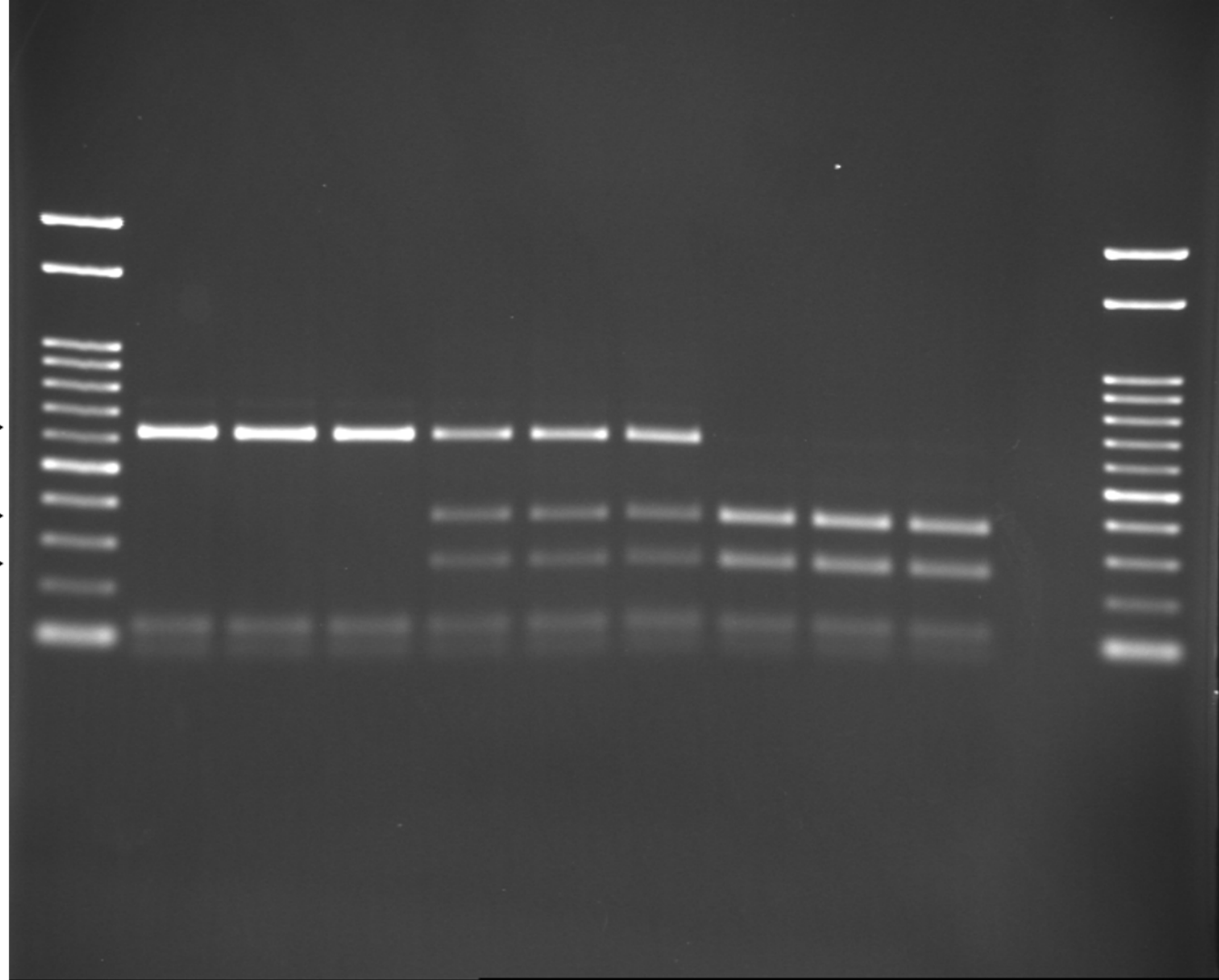

2000 bp

1000 bp

500 bp

100 bp

1 2 3 4 5 6 7 8 9 10 11 12

Supplement: S1 Raw images — (Lanes 1 and 12) Molecular size marker. (Lanes 2–4) D. rerio gave a single fragment of 617 bp. (Lanes 5–7) The hybrids between D. rerio female and D. nigrofasciatus male possessed three fragments of 617, 365 and 252 bp. (Lanes 8–10) D. nigrofasciatus gave two fragments of 365 and 252 bp. (Lane 11) Negative control. (PDF) [file pone.0233885.s001.pdf]
